# Simplifying the detection of *MUTYH *mutations by high resolution melting analysis

**DOI:** 10.1186/1471-2407-10-408

**Published:** 2010-08-05

**Authors:** Isabel López-Villar, Rosa Ayala, Jan Wesselink, Juan Diego Morillas, Elena López, José Carlos Marín, José Díaz-Tasende, Sara González, Luis Robles, Joaquín Martínez-López

**Affiliations:** 1 Department of Molecular Biology, 12 De Octubre University Hospital, Madrid, E-28041, Spain; 2 CBM, UAM Universidad Autónoma de Madrid, Spain; 3 Department of Gastroenterology 12 De Octubre University Hospital, Madrid, E-28041, Spain; 4 Biotechnology Programme, Spanish National Cancer Research Centre, Madrid, Spain; 5 Institut Català d'Oncologia ICO, Barcelona, Spain; 6 Department of Medical Oncology 12 De Octubre University Hospital, Madrid, E-28041, Spain

## Abstract

**Background:**

*MUTYH*-associated polyposis (MAP) is a disorder caused by bi-allelic germline *MUTYH *mutation, characterized by multiple colorectal adenomas. In order to identify mutations in *MUTYH *gene we applied High Resolution Melting (HRM) genotyping. HRM analysis is extensively employed as a scanning method for the detection of heterozygous mutations. Therefore, we applied HRM to show effectiveness in detecting homozygous mutations for these clinically important and frequent patients.

**Methods:**

In this study, we analyzed phenotype and genotype data from 82 patients, with multiple (>= 10) synchronous (19/82) or metachronous (63/82) adenomas and negative *APC *study (except one case). Analysis was performed by HRM-PCR and direct sequencing, in order to identify mutations in *MUTYH *exons 7, 12 and 13, where the most prevalent mutations are located. In monoallelic mutation carriers, we evaluated entire *MUTYH *gene in search of another possible alteration. HRM-PCR was performed with strict conditions in several rounds: the first one to discriminate the heteroduplex patterns and homoduplex patterns and the next ones, in order to refine and confirm parameters. The genotypes obtained were correlated to phenotypic features (number of adenomas (synchronous or metachronous), colorectal cancer (CRC) and family history).

**Results:**

*MUTYH *germline mutations were found in 15.8% (13/82) of patients. The hot spots, Y179C (exon 7) and G396D (exon 13), were readily identified and other mutations were also detected. Each mutation had a reproducible melting profile by HRM, both heterozygous mutations and homozygous mutations. In our study of 82 patients, biallelic mutation is associated with being a carrier of ≥10 synchronous polyps (p = 0.05) and there is no association between biallelic mutation and CRC (p = 0.39) nor family history (p = 0.63). G338H non-pathogenic polymorphism (exon 12) was found in 23.1% (19/82) of patients. In all cases there was concordance between HRM (first and subsequent rounds) and sequencing data.

**Conclusions:**

Here, we describe a screening method, HRM, for the detection of both heterozygous and homozygous mutations in the gene encoding *MUTYH *in selected samples of patients with phenotype of MAP. We refine the capabilities of HRM-PCR and apply it to a gene not yet analyzed by this tool. As clinical decisions will increasingly rely on molecular medicine, the power of identifying germline mutations must be continuously evaluated and improved.

## Background

MAP is a disorder caused by bi-allelic germline *MUTYH *mutations, characterized by multiple colorectal adenomas. Mutations are distributed over the *MUTYH *locus, most of the changes found are missense mutations, of which Y179C and G396D, located in *MUTYH *exons 7 and 13 respectively, pose approximately 73% of mutations found in western populations [1,2]. Analysis of 82 selected patients was performed by HRM-PCR and direct sequencing, in order to identify mutations in *MUTYH *exons 7, 12 and 13. We also analyzed the prevalence of a non-pathogenic polymorphism, located in exon 12: G338H [3]. We evaluated the ability of HRM [2] for genotype at specific positions and also unknown mutations. HRM has become an alternative for screening for molecular diagnosis. In normal application HRM-PCR, discriminates heteroduplexes, which melt at lower temperatures than homoduplexes [2]. However, mutant homozygotes have been reported on the germline *MUTYH *gene [1]. The importance of identifying these mutations is based on the fact that MAP represents a syndrome predisposing colorectal cancer with an autosomal recessive pattern. In fact, these mutations are responsible for as many as 40% of cases with attenuated familial adenomatous polyposis without mutations in the *APC *gene. In this regard, downstream assays are limited in clinical decision-making and outcome [4-6]. Thus, this application provides a platform for detection of germline mutations in *MUTYH *gene. HRM-PCR was performed in several rounds with specific primers: the first one discriminating heteroduplex and homoduplex patterns and the next ones, in order to refine the tight conditions. Biallelic *MUTYH *mutations have also been found to be appended with 93-fold excess risk of colorectal cancer, with practically complete penetrance by 60 years of age [1].

## Methods

### Patients

This study was a collaboration amongst 3 research Departments from *12 De Octubre *Hospital: Gastroenterology, Oncology and Molecular Biology. In this study, we analyzed phenotype and genotype data from 82 patients, with multiple (>= 10) synchronous (19/82) or metachronous (63/82) adenomas and negative *APC *study (except one case). The patients, listed in Table 1, were collected and analyzed for the presence of *MUTYH *mutations. Included were average ages at presentation of polyps (ranging from 17 to 88), type of polyps (≥10) metachronous (63/82) or synchronous (19/82), family history and, if any, location, stage and age at presentation of colorectal cancer (CRC). In this case, the occurrence of CRC staging was classified according to the modified Astler-Coller guide and immunohistochemistry was performed to rule out deficient mismatch repair (MMR) proteins: *MLH1, MSH2, MSH6 *and *PMS2*. All subjects signed informed consent forms after collection of blood specimens and this study was approved by the Internal Ethics Committee of *12 De Octubre *University Hospital.

**Table 1:** Phenotypic features of 82 patients

Type of Adenomas		CRC		Family history	
Patients with multiple ≥ 10) polyps synchronous	Patients with multiple (≥ 10) polyps metachronous	Yes	No	Yes	No
23.2% 19/82	76.8% 63/82	48.8% 40/82	51.2% 42/82	34.1% 28/82	65.9% 54/82
Mean age at presentation 63.7 years	Mean age at presentation 61.0 years	Mean age at presentation 57.1 years			

### DNA samples

We extracted genomic DNA from peripheral blood of 82 selected patients from the Endoscopy Department of the *12 De Octubre *Hospital. We isolated genomic DNA with the automatic Blood Extraction Kit based on magnetic bed technology according to manufacturer protocol (Masswell Promega, Madison, WI USA). We measured DNA concentration using a NanoDrop 1000 spectrophotometer (NanoDrop Technologies Inc., Wilmington, DE, USA) and we diluted the samples to a final concentration of 10 ng/μl.

### Primers used for HRM-PCR, identification of the assay conditions

We evaluated 3 amplicons corresponding to *MUTYH *exons 7, 12 and 13. The primers used for amplification of *MUTYH *gene segments via HRM-PCR are listed in Table 2, along with the amplicon size_. _The primers were designed to be annealed at 60°C using Primer Express software (Applied Biosystems, Foster City, CA) to calculate melting temperature (T_m_). The primers used for amplification of the remaining *MUTYH *gene are listed in Table 3.

**Table 2:** *MUTYH *HRM and sequencing primer sequences

Exon	Primer name	Sequence	Amplicon size (base pairs)
7	7F	5'-GGGACTGACGGGTGATCTCT-3'	186 bp
	7R	5'-TTGGAGTGCAAGACTCAAGATT-3'	

12	12F	5'-AGCCCTCTTGGCTTGAGTA-3'	297 bp
	12R	5'-TGCCGATTCCCTCCATTCT-3'	

13	13F	5'-AGGGCAGTGGCATGAGTAAC-3'	296 bp
	13R	5'-GGGTCAAGGGGTTCAAATAG-3'	

**Table 3:** *MUTYH *sequencing primer sequences

Exon	Primer name	Sequence	Amplicon size (base pairs)
1	1F	5'-GCGGTGTACAACGGAACTTG-3'	292 bp
	1R	5'-ATCCCCGACTGCCTGAACC-3'	
2	2F	5'-CTGCTTTGGCTGGGTCTTT-3'	262 bp
	2R	5'-CGCACCTGGCCCTTAGTAAG-3'	
3	3F	5'-CTGCTGTGTCCCAAGACC-3'	299 bp
	3R	5'-CAACCCCAGATGAGGAGTTAGG-3'	
4	4F	5'-GACCTACCATGGAGAAGACG-3'	252 bp
	4R	5'-GGGTTGGCATGAGGACACTG-3'	
5	5F	5'-GGGCAGGTCAGCAGTGTC-3'	189 bp
	5R	5'-TACACCCACCCCAAAGTAGA-3'	
6	6F	5'-TACTTTGGGGTGGGTGTAGA-3'	185 bp
	6R	5'-AAGAGATCACCCGTCAGTCC-3'	
8	8F	5'-CCAGGAGTCTTGGGTGTCTT-3'	240 bp
	8R	5'-AGAGGGGCCAAAGAGTTAGC-3'	
9	9F	5'-AACTCTTTGGCCCCTCTGTG-3	196 bp
	9R	5'-GAAGGGAACACTGCTGTGAAG-3'	
10	10F	5'-GTGCTTCAGGGGTGTCTGC-3'	262 bp
	10R	5'-TGTCATAGGGCAGAGTCACTCC-3'	
11	11F	5'-TAAGGAGTGACTCTGCCCTATG-3'	251 bp
	11R	5'-GCCAAGAGGGCTTTAGGG-3'	
14	14F	5'-TTGGCTTTTGAGGCTATATCC-3'	256 bp
	14R	5'-CATGTAGGAAACACAAGGAAGTA-3'	
15	15F	5'-TGAAGTTAAGGGCAGAACACC-3'	207 bp
	15R	5'-GTTCACCCAGACATTCGTTAGT-3'	
16	16F	5'-AGGACAAGGAGAGGATTCTCTG-3'	298 bp
	16R	5'-AGACCCCCATCTCAAAAA-3'	

First we designed primers to flank the coding regions of *MUTYH *exons and analyzed each amplicon in order to ensure that it contained a single melting domain using the Poland program http://www.biophys.uni-duesseldorf.de/local/POLAND/poland.html. We included intronic SNPs, close to the exon boundary, always respecting the condition of amplicons under 300 bp. This was possible as the size of *MUTYH *exons allowed this design. All primer sequences were analyzed (http://genome.ucsc.edu/cgi-bin/hgPcr) to adjust the likelihood that interferences would not co-amplify with the target sequence melting curves.

In monoallelic mutation carriers, we evaluated by HRM-PCR and sequencing entire *MUTYH *gene in search of another possible alteration.

### HRM-PCR

This was performed and monitored in a Light Cycler 480 machine (Roche Diagnostics, Penzberg, Germany). HRM-PCR was performed in several rounds, the first one discriminating heteroduplex and homoduplex patterns. First round: we amplified DNA fragments (*MUTYH *exons 7, 12 and 13) from 5-10 ng genomic DNA. Full reactions contained final concentrations of reagents as follows: 2 mM MgCl_2_, 0.12 μΜ forward and reverse primers listed in Table 2, 2 × HRM Master (containing ResoLight dye) and DNA. Full HRM-PCR cycling and melting conditions were as follows: 95°C,10 min; 40 cycles of (95°C, 10 s; 60°C fluorescence reading, 10 s; 72°C, 15 s) then melting of (95°C, 1 min; 40°C, 1 min; 60°C, 1 s; 95°C 25 acquisitions per °C). We performed several rounds in each exon, using re-extracted DNA samples and the same concentration of arrangements and conditions. For reproducibility, the analyst repeated the procedures on three different days.

HRM analyses were performed on the latest Software (v.1.5) and the results were blinded to the sequencing data. The melting curves were normalized and temperature shifted, to permit samples to be compared. Significant differences in fluorescence from the horizontal baseline, previously selected, were indicative of mutations. For an evaluation of the mutation detection obtained via HRM-PCR, mutation-containing DNA was amplified using the full HRM-PCR program. In order to remove background fluorescence we subjected raw fluorescence data to normalization and temperature shifting. The probability that the observed melting temperature (Tm) separation of alternative homozygotes was assessed using the nonparametric Mann-Whitney test. We started HRM analyses with sensitivity 0.3 and it was adjusted in the following rounds.

In monoallelic mutation carriers, we evaluated entire *MUTYH *gene in search of another possible alteration, by HRM-PCR: Full reactions contained final concentrations of reagents as follows: 2 mM MgCl_2_, 0.12 μΜ forward and reverse primers listed in Table 3, 2 × HRM Master (containing ResoLight dye). Full HRM-PCR touch-down cycling and melting conditions were as follows: 95°C,10 min; 40 cycles of (95°C, 10 s; (65°C-58°C step size 0,5°C and step delay 1 cycle, fluorescence reading), 10 s; 72°C, 15 s) then melting of (95°C, 1 min; 40°C, 1 min; 60°C, 1 s; 95°C 25 acquisitions per °C). The analyst repeated the procedures on three different days.

### DNA sequencing

We sequenced all samples, to evaluate the detection of *MUTYH *mutations and correlate these with HRM results. Amplifying DNA fragments from 50-100 ng. Reactions contained final concentrations of reagents as follows: 0.8 mM MgCl_2_, 0.7 μΜ forward and reverse primers listed in Table 2 and Table 3, 1 × Taq polymerase fast start (Roche Diagnostics, Penzberg, Germany) and DNA. PCR cycling and conditions were: 95°C, 10 min; 35 cycles of (95°C, 30 s; 60°C, 30 s; 72°C, 30 s) and 72°C, 10 min. PCR products were purified with ExosapIT (GE Healthcare) followed by sequencing reaction with Big Dye Terminator v3.1 (Applied Biosystems, Foster City, CA) according to the manufacturer's protocol. The sequencing products were purified using AutoSeq G-50 Dye Terminator (GE Healthcare) before running on a 3130 Genetic Analyser (Applied Biosystems, Foster City, CA).

### Downstream assays

HRM genotyping of *MUTYH *gene was performed at the *12 De Octubre *Hospital for genomics genotyping Core Facility.

### Statistical analysis

The statistical program used for the analysis was SPSS version 15.0. The association between categorical variables was made by χ2 test and Fisher exact test and odds ratio was used to measure the strength of the association. The association between continuous variables was conducted by comparing means using the T test of Student or Mann-Whitney. The significance level was at <or equal to 0.05.

## Results

*MUTYH *germline mutations were found in 15.8 percent, i.e. 13/82 of the patients. Biallelic *MUTYH *germline mutations were found in 8.5%, i.e. 7/82 which also showed an attenuated polyposis phenotype. Monoallelic *MUTYH *germline mutations were found in 7.3% i.e. 6/82 of the patients. In monoallelic mutation carriers, we performed HRM-PCR and sequenced entire *MUTYH *gene in search of another possible alteration: another mutation was detected in two cases (2/8 entire gene studied cases).

The *hot spots*: Y179C (exon 7) and G396D (exon 13) were found, as well as the five mutations already reported E410GfsX43 (exon 13), R426C (exon 13), R354GfsX40 (exon 12), V232F (exon 9) and V22M (exon 2).

G338H polymorphism (exon 12) was also analyzed in the cohort of 82 subjects and it was found in 23.1%, i.e. 19/82.

The two most frequent mutations reported to date [7] (Y179C and G396D) were detected in quite a number of the mutated cases, the frequency of these alleles being 61.5%, i.e. 8/13. Amongst the other mutations found [8], the E410GfsX43 (exon 13) accounted for 23.0%, i.e. 3/13, of the mutant alleles reported (Table 4).

**Table 4:** *MUTYH *mutation prevalence for 82 patients with multiple adenomas (≥10), determined via HRM-PCR and via sequencing

		Wild type (%)	Homozygous (%)	Heterozygous (%)
Mutations founded	Y179C (exon 7)	96.4 (79/82)	1.2 (1/82)	2.4 (2/82)
	G396D (exon 13)	92.7 (76/82)	1.2 (1/82)	6.1 (5/82)
	E410GfsX43 (exon 13)	95.1 (78/82)	0.0 (0/82)	4.9 (4/82)
	R426C (exon 13)	97.6 (80/82)	0.0 (0/82)	2.4 (2/82)
	V232F (exon 9)	98.8 (81/82)	0.0 (0/82)	1.2 (1/82)
	V22M (exon 2)	98.8 (81/82)	0.0 (0/82)	1.2 (1/82)
	R354GfsX40 (exon 12)	98.8 (81/82)	0.0 (0/82)	1.2 (1/82)
Polymorphism tested	G338H (exon 12)	76.8 (63/82)	0.0 (0/82)	23.2 (19/82)

Phenotypic and genotypic features in the 7 patients carriers of biallelic *MUTYH *germline mutations are shown in Table 5, collecting the variables: type of detected mutation, type of adenomas, family history, analysis of G338H non-pathogenic polymorphism and, in the case of colorectal cancer (CRC), stage and age at presentation. In our study biallelic mutation is associated with being a carrier of synchronous polyps (p = 0.05) and there is no association between biallelic mutation and colorectal cancer (p = 0.39) nor family history (p = 0.63).

**Table 5:** Phenotypic and genotypic features in the 7 patients carriers of biallelic *MUTYH *germline mutations

	Patients with biallelic germline mutations	Number of adenomas (≥10) synchronous or metachronous	CRC	Family history	Polymorphism G338H
**Patient 1**	Y179C (exon 7) homozygous	Polyps synchronous Age 58	No	No	No
**Patient 2**	V22M (exon 2) E410GfsX43 (exon 13) double heterozygote	Polyps synchronous Age 42	No	Yes	No
**Patient 3**	R426C (exon 13) E410GfsX43 (exon 13) double heterozygote	Polyps synchronous	Yes Age diagnosis Astler-Coller A	Yes 50	No
**Patient 4**	R426C (exon 13) R354GfsX40 (exon 12) double heterozygote	Polyps metachronous Age 67	No	No	Yes
**Patient 5**	Y179C (exon 7) E410GfsX43 (exon 13) double heterozygote	Polyps synchronous	Yes Age diagnosis Astler-Coller B1	No 63	No
**Patient 6**	G396D (exon 13) V232F (exon 9) double heterozygote	Polyps synchronous Age 82	No	No	No
**Patient 7**	G396D (exon 13) homozygous	Polyps synchronous Age 58	No	No	No

Phenotypic and genotypic features in the 6 patients carrying monoallelic *MUTYH *germline mutations show that monoallelic mutations have no association with being a carrier of synchronous polyps (p = 0.66) nor colorectal cancer (p = 0.79) and also no association between monoallelic mutations and family history (p = 0.38) (Table 6).

**Table 6:** Phenotypic and genotypic features in the 6 patients carriers of monoallelic *MUTYH *germline mutations

	Patients with monollelic germline mutations	Number of adenomas (≥ 10) synchronous or metachronous	CRC	Family history	Polymorphism G338H
**Patient 1**	Y179C (exon 7) heterozygote	Polyps synchronous Age 43	No	Yes	No
**Patient 2**	G396D (exon 13) heterozygote	Polyps metachronous Age 42	Yes Age diagnosis Astler-Coller A	Yes 40	No
**Patient 3**	R426C (exon 13) heterozygote	Polyps synchronous Age 74	No	No	No
**Patient 4**	G396D (exon 13) heterozygote (*MUTYH *and *APC *genes are mutated) In *APC *gene L126S heterozygous	Polyps synchronous Age 51	No	Yes	Yes
**Patient 5**	G396D (exon 13) heterozygote	Polyps metachronous Age 70	No	Yes	No
**Patient 6**	E410GfsX43 (exon 13) heterozygote	Polyps metachronous Age 39	No	Yes	No

In relation to the 19 carriers of G338H polymorphism [9] we found no association with any of the following variables: synchronous adenomas (p = 0,76), CRC (p = 0,79) and family history (p = 0,11) in this selected group. (Table 7).

**Table 7:** Phenotypic and genotypic features in the 19 patients carriers of polymorphism G338H (exon 12)

Type of adenomas		CRC		Family history In 9 cases no information was available	
Patients with multiple (≥ 10) polyps synchronous	Patients with multiple (≥ 10) polyps metachronous	Yes	No	Yes	No
31,6% 6/19	68,4% 13/19	52,6% 10/19	47,4% 9/19	26,3% 5/19	26,3% 5/19

To assess the ability to differentiate between alleles, by high resolution melting technique [10-12], PCR products (first and subsequent rounds) were processed by latest Software (v.1.5) and the results were blinded to the sequencing data [13-15]. First round HRM-PCR (exon 7 with sensitivity of 0.3 and exon 13 with sensitivity of 0.45) discriminated between heteroduplex, wild homoduplex and mutant homoduplex patterns. Several rounds were carried out, as a confirmatory method and to set up the appropriate sensitivity of each exon. For reproducibility, the analyst repeated the procedures on three different days, with equivalent results. We did not have any false calls.

**Figure 1:**
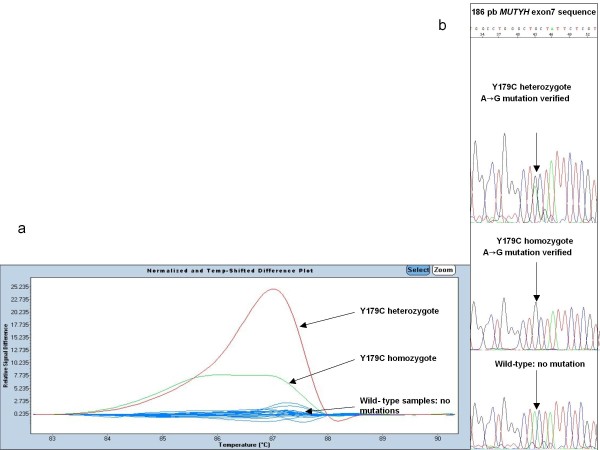
**Different plot and sequence traces for *MUTYH *exon 7 mutations**. (**a**) The melting profile with sensitivity 0,3. The figure shows that mutations were clearly distinct from the wild type controls. Melt curves of each mutation (red: Y176C heterozygote, green: Y179C homozygote were plotted against the wild types (blue). (**b**) Sequencing electropherograms show a Y179C heterozygous mutation, a Y179C homozygous mutation and a wild type.

In relation to *MUTYH *exon 7, the results are listed in Figure 1: mutations were clearly distinct from the wild type controls in the first round. Melt curves of each mutation heterozygote, homozygote, were plotted against the wild types.

**Figure 2:**
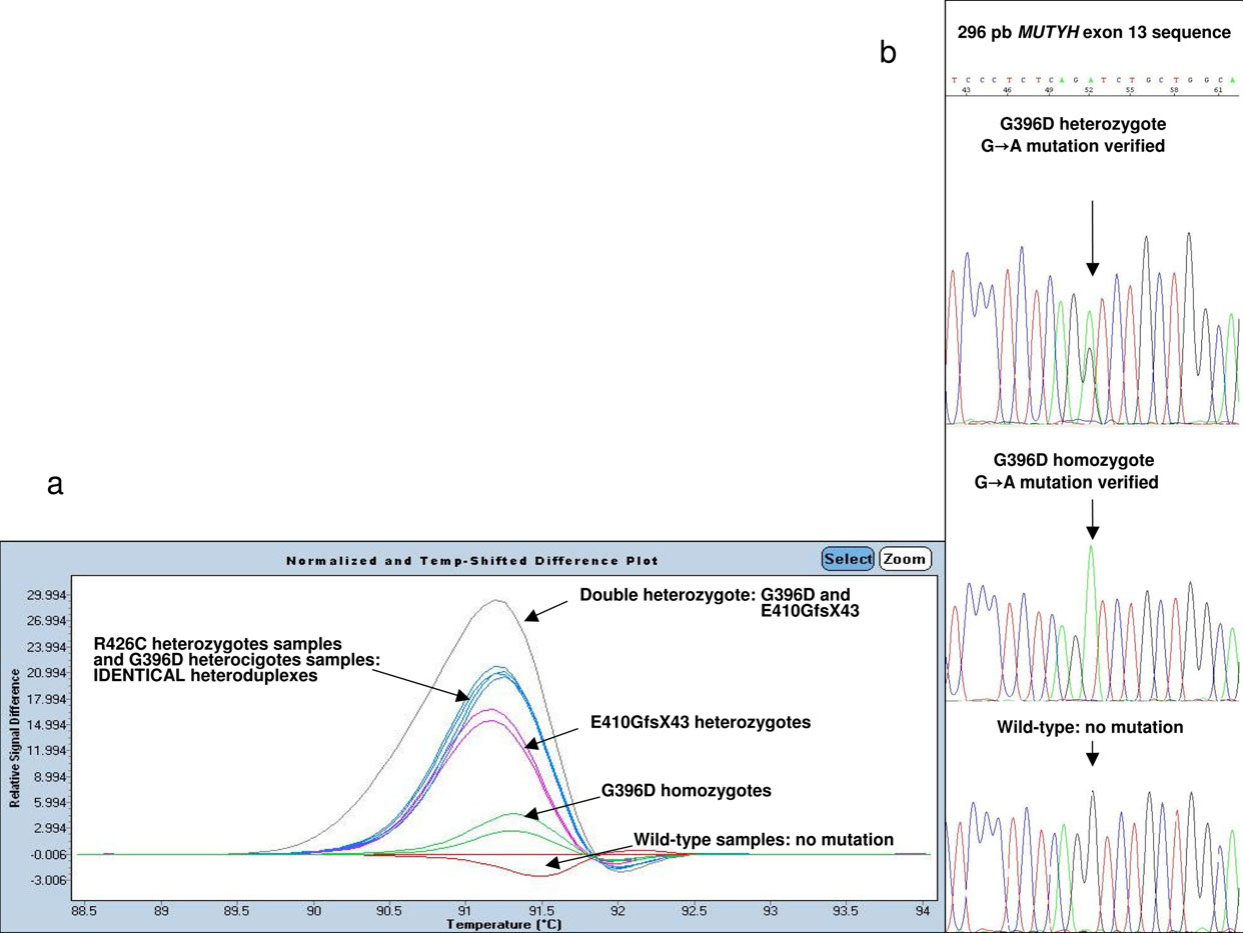
**Difference plot and sequence traces for *MUTYH *exon 13 mutations**. (**a**) Difference plot and sequence traces for *MUTYH *exon 13 mutations. (a) The melting profile with sensitivity 0,45. The figure shows that mutations were clearly distinct from the wild type controls. R426C heterozygote and G396D heterozygote have identical heteroduplex melting patterns. Melt curves of each mutation (green: G396D homozygotes, pink: E410GfsX43 heterozygotes, blue: R426C heterozygotes and G396D heterozygotes, grey: double heterozygote G396D and E410GfsX43 against the wild type (red). (**b**) Sequencing electropherograms show a G396D heterozygous mutation, a G396D homozygous mutation and a wild type.

Similar to the previous analysis, Figure 2 includes different plot and sequence traces for *MUTYH *exon 13; First round: mutations clearly distinct from the wild type controls; but R426C heterozygote and G396C heterozygote have identical heteroduplex melting patterns (this differed in the subsequent sequencing).

**Figure 3:**
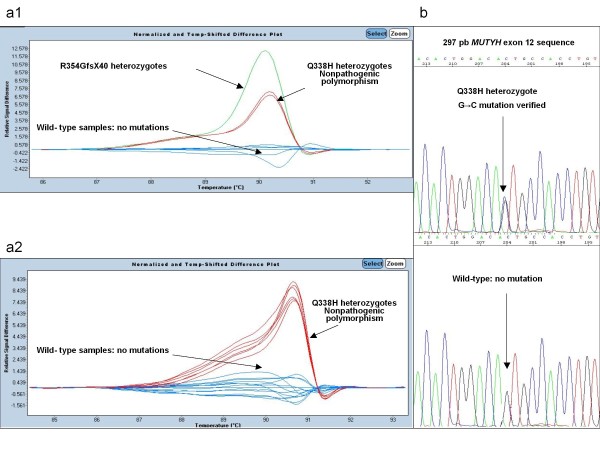
**Different plot and sequence traces are indicated for *MUTYH *exon 12 mutations**. (**a1**) (**a2**) The melting profile with sensitivity 0,3. The figures shows that mutations were clearly distinct from the wild type controls. Melt curves of each mutation, green: R354GfsX40 heterozygotes, red: G338H non-pathogenic heterozygotes polymorphism against the wild type (blue). (**b**) Sequencing electropherograms show a G338H heterozygous polymorphism and a wild type.

In Figure 3 different plot and sequence traces are indicated for *MUTYH *exon 12 mutations.

In all cases there was concordance between HRM and sequencing data. (Figures 1, 2 and 3).

## Discussion

We found *MUTYH *germline mutations in 15.8 percent, i.e. 13/82 of the patients with multiple adenomas. This percentage is obtained from the analysis of three exons, 7,12 and 13 in the 82 cases and the posterior analysis of entire *MUTYH *gene in monoallelic mutation carriers. We believe it feasible that this percentage would be slightly increased if we analyze the entire gene in the 82 cases[1]. We gathered information from the most prevalent hot spots at that moment in Spain.

The average age at diagnosis of CRC in studied families was 57 years (ranging from 24 to 86).

In contrast, classical FAP (familial adenomatous polyposis) patients, as is described in the literature [16-18], show a CRC onset 10 years earlier than MAP (mean age at presentation 39 *versus *53 respectively)[16].

It appears that disease symptoms in the 7 MAP patients are not as severe as those observed in *APC *driven FAP. The mutations found along the *MUTYH *gene were as follows: the hot spots Y179C and G396D; and as well as the five mutations already reported. The highest prevalence of mutations corresponds to the *hot spots *mentioned above.

In the study of G338H non-pathogenic polymorphism (exon 12) our data indicate no association with attenuated familial adenomatous polyposis (MAP) in this selected group.

By discriminating between distinctive individuals based on their featured high-resolution melting curves[11], one of the most challenging tasks is the discrimination of amplicons differing by homoduplexes. HRM is a rapid and informative toll, but requires specific primers and refines the sensitivity post-PCR analysis in case of differing homoduplexes. We demonstrate that this tool, HRM, can also be used to detect homozygous mutations in MAP patients. In this way, homozygous mutations show a mutated homoduplex by HRM which is perfectly discriminated against wild type homoduplex and against mutated heteroduplex. (Figures 1, 2 and 3).

A HRM pre-screening assay of exons 7, 12 and 13 could be integrated into the laboratory routine, so that a large number of samples can be screened for identification of samples of interest.

We estimate that by using HRM as a screening method, the number of sequencing reactions requiring *MUTYH *mutation detection can be reduced by up to 80% thus resulting in substantial time and cost savings[13]. Sequencing is reduced, because once HRM-PCR is optimized for this gene, only abnormal patterns are sequenced. This shows the high resolving power of HRM, compared with direct sequencing[14]. Hence HRM can be used to combat unknown mutation-detection technologies. We refine the capabilities of HRM-PCR and apply it to a gene not yet analyzed by this method[15].

The combination of real-time PCR and high-resolution melting curve analysis provides an approach to successfully scan exons 7, 12 and 13 of *MUTYH *gene for these clinically important and frequent mutations.

## Conclusions

In conclusion, we describe a screening method HRM, to detect heterozygous and homozygous mutations in the gene encoding *MUTYH *in selected samples of patients with phenotype of MAP. As clinical decisions will increasingly rely on molecular medicine, the power of identifying germline mutations must be continuously evaluated and improved.

## Competing interests

The authors declare that they have no competing interests.

## Authors' contributions

ILV, JW, RA, EL, SG and JML experimental design, project setup and manuscript preparation; JDM, JCM, JDT and LR clinical considerations and rationale. All authors read and approved the final manuscript.

## Pre-publication history

The pre-publication history for this paper can be accessed here:

http://www.biomedcentral.com/1471-2407/10/408/prepub
